# Supplementary material for: Metabolomic characteristics of aerobic and resistance exercise modes
Source: PLoS One. 2025 Dec 12;20(12):e0338814. doi: 10.1371/journal.pone.0338814 (PMC12700455; doi:10.1371/journal.pone.0338814)
Supplement: S1 File — (DOCX) [file pone.0338814.s001.docx]

**Supporting Information**

**Sample collection**

Urine samples ere collected respectively 30 minutes before exercise, 30 minutes after exercise and 24 hours after recovery. A total of 60 urine samples were collected, aliquoted and stored at -80 °C until analysis.

**Metabolite Extraction**

Urine samples stored at -80°C were thawed at room temperature and then centrifuged at 4000rcf for 2-3min. Take 300μl of the supernatant and add 1200μl of 4°C pre-cooled MeOH: ACN (1:1, v/v). After vortex mixing, centrifuge at 4℃, 13000rcf for 20min. Take 1ml of the supernatant and blow dry slowly with a nitrogen blower. Add 150μl ACN: H2O (1:1, v/v) to re-dissolve. Vortex and mix well and sonicate in water bath for 2min. Vortex and mix again, centrifuge at 4℃, 12000rcf for 10min. 80μl of the supernatant was taken in a mass spectrometry injection vial. Blank samples were prepared by Milli-Q ultrapure water, and quality control (QC) samples were prepared by mixing 10μl of each sample, and one QC was inserted in every 10 samples for evaluating the system stability during the experiment.

**LC‑MS/MS Analysis**

For untargeted metabolomic analysis, UPLC-QTOF-MS analysis was performed on a Triple TOF 5600 mass spectrometer (SCIEX, USA) coupled to a Agilent 1290 Infinity II Ultra Performance Liquid Chromatography system (Agilent, USA) in both positive and negative ion modes. The mass scanning range was m/z 50-1000 in full data storage mode. The drying gas temperature was set at 600 °C . The ion spray voltage was 5500 V in positive mode and -4500 V in negative mode.

Atomization gas pressure, auxiliary heating gas pressure and curtain gas pressure in both ionization modes were set at 60, 55 and 35 psi, respectively. The separation was performed using an Acquity UPLC BEH Amide column (100 × 2.1 mm, 1.7 µm, Waters, USA) in binary gradient mode. The mobile phases in different ionization modes were as follows: (A) 10 mM ammonium format and 0.1% (v/v) formic acid in water and (B) pure ACN in positive mode. The flow rate was 300 μL·min-1. The gradient was programmed as follows: initial conditions of 95% of mobile phase B followed by a linear gradient from 95% to 65% of mobile phase B in 13 min and then from 65% to 40% in 2 min; the 40% elution of mobile phase B was held for 2 min and then, a gradient of 0.1 min was used to return to the initial conditions, which were held for an additional 2.9 min. Column temperatures was set to 40 °C and the injection volume used was 5 µl. Samples were placed in randomized order. One QC sample and one blank vial were run after each of the 10 urine samples.

**Metabolomics Total Ion Flow Chromatography (TIC)**

S1_Fig. shows the TIC overlay of the QC samples in positive and negative ion mode. As shown, the retention time and peak area of the QC sample overlap well, indicating good instrument stability and reliable results.

**Principal component analysis (PCA)**

S1_Fig2. shows the PCA plots. QC samples are incorporated to monitor and ensure the stability of the entire analytical workflow. QC samples cluster tightly within the PCA plots.
