# Supplementary figures and images for: Metabolomic characteristics of aerobic and resistance exercise modes
Source: PLoS One. 2025 Dec 12;20(12):e0338814. doi: 10.1371/journal.pone.0338814 (PMC12700455; doi:10.1371/journal.pone.0338814)

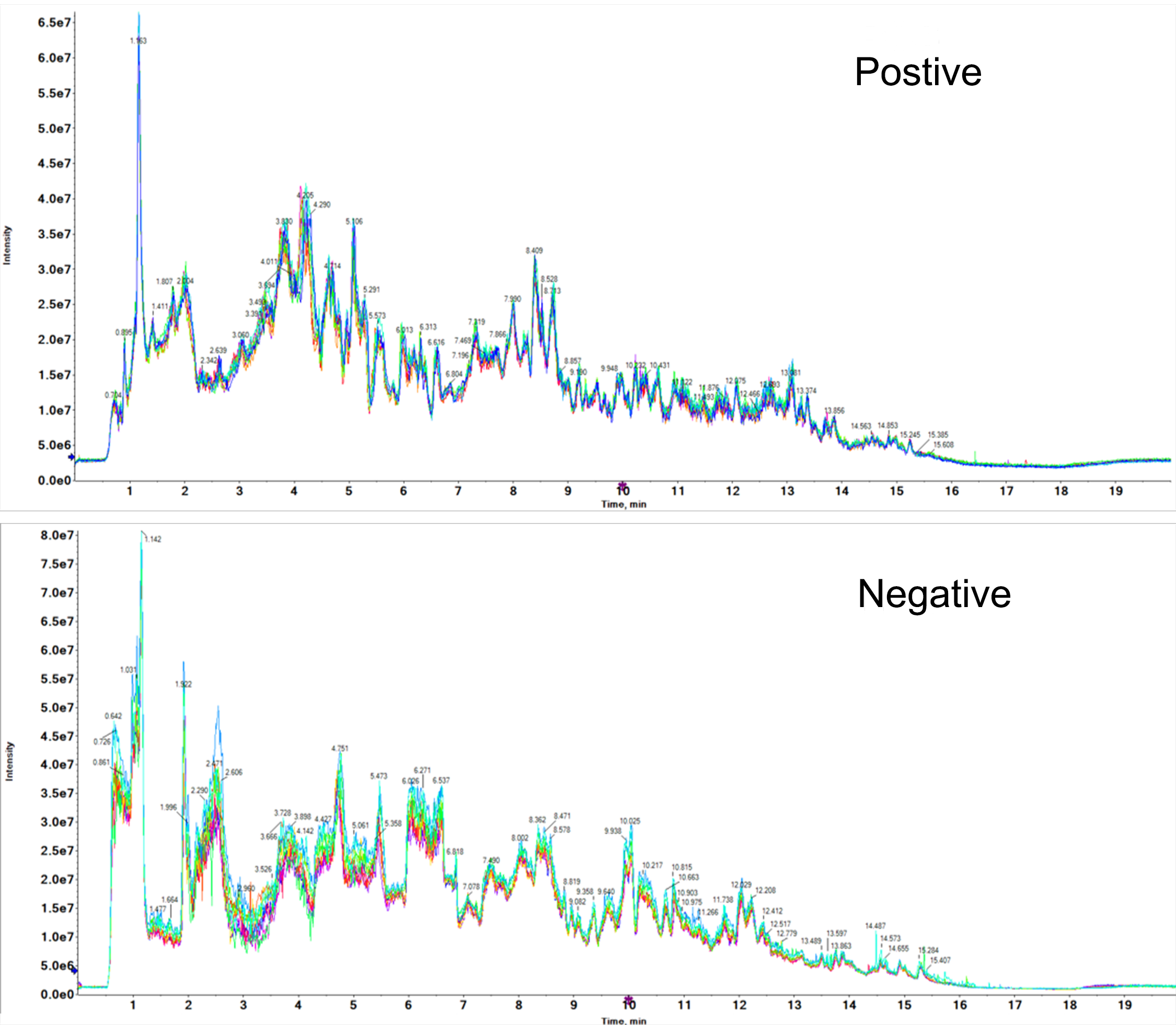

Supplement: S1 Fig — (TIF) [file pone.0338814.s002.tif]

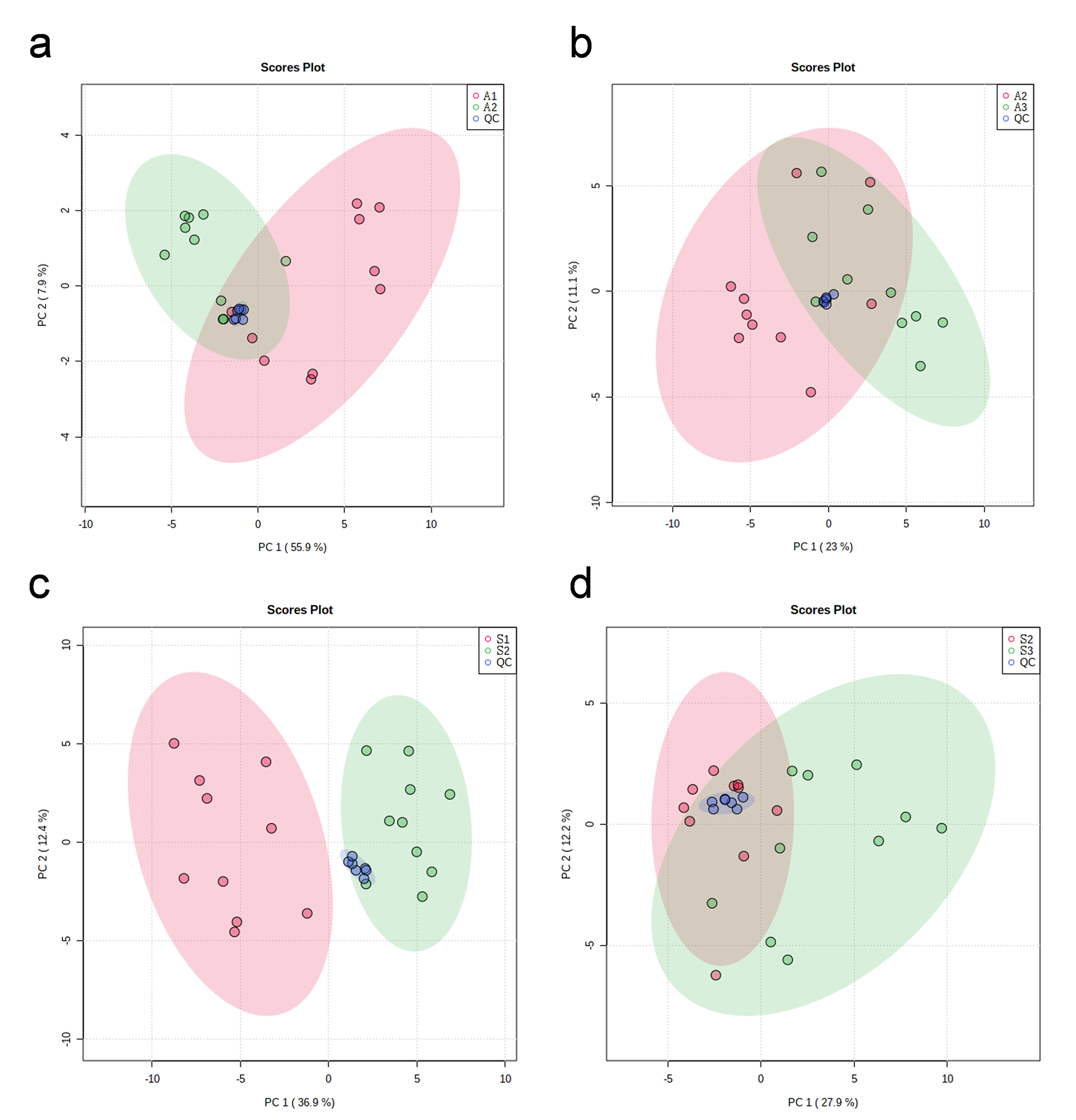

Supplement: S2 Fig — (TIF) [file pone.0338814.s003.tif]
