# Supplementary material for: Metabolomic characteristics of aerobic and resistance exercise modes
Source: PLoS One. 2025 Dec 12;20(12):e0338814. doi: 10.1371/journal.pone.0338814 (PMC12700455; doi:10.1371/journal.pone.0338814)
Supplement: Attachment — Submitted filename: PONE-D-25-28714.docx [file pone.0338814.s004.docx]

The study titled “*Metabolomic Characteristics of Aerobic and Resistance Exercise Modes*” by *Junjie Kuang* et.al explored significant changes in few metabolites such as amino acids in aerobic and resistance exercise persons using metabolomics approach.

The study makes an ordinary contribution to the understanding of the physical activity associated with metabolic regulations. This present research work is interesting and publishable after addressing some important concerns.

1. Novelty is limited. Author should explain exact hypothesis mechanism behind this study.
2. How this present study is translational to human pathophysiology and associated to mankind.
3. Why author explaining by focus on the amino acid metabolism instead of others like sungar and associated phosphate because these all are contributing factors in the muscle’s physiology.
4. Authors should represent the data accurately.
5. I would suggest to see some metabolomics articles that can be helpful for this study data representation.
6. The metabolites table is incorrectly represented i.e., RT. Please check carefully.
7. Author should provide the clearer figures and reasonable biology of the acquired data.
8. Author should provide the biology of metabolites associated with metabolic pathway or cycle that can be better for this theme of study.
9. Author should provide the more details of the study outcome in discussion section.
10. Elimination of these deficiencies can be understandable and publishable.
