# Supplementary material for: Metabolomic characteristics of aerobic and resistance exercise modes
Source: PLoS One. 2025 Dec 12;20(12):e0338814. doi: 10.1371/journal.pone.0338814 (PMC12700455; doi:10.1371/journal.pone.0338814)
Supplement: Attachment — Submitted filename: Response to Reviewers.docx [file pone.0338814.s006.docx]

**Response to Reviewers**

1 Summarizing the implications of the research as follows: This study aims to outline the metabolic responses induced by acute aerobic and resistance training in healthy male college students. It identifies indicators reflecting physiological status and exercise efficacy.
2 The innovative points of this study lie in: Collect urine samples and employe LC-MS-based metabolomic analysis to investigate the effects of different exercise modes and time points before and after a single exercise session on metabolic characteristics.
3 Hypothesis mechanism: Different types of acute exercise sessions elicit distinctly different urinary metabolome responses. Specifically, we anticipate that aerobic exercise will significantly alter levels of fatty acids and tricarboxylic acid cycle intermediates by activating mitochondrial oxidative function. In contrast, resistance exercise may lead to a sharp increase in lactate and purine metabolites through anaerobic glycolysis.
  a) We fully acknowledge that the limited sample size (n=10) may impact statistical power. As a preliminary mechanistic exploratory study, our primary target is to screen key metabolites or pathways to generate hypotheses for follow-up large-scale confirmatory research. Many metabolomics studies have adopted similar sampling size strategies. (e.g. Floegel, A., et al. (2013), Barton, R. H., et al. (2010)). Through rigorous experimental controls and longitudinal sampling, the study has somewhat minimised confounding factors. We have reinforced this point in the “Limitations” section of the manuscript.
  b) We fully acknowledge that the inability to elucidate gender-specific metabolic responses constitutes one of the core limitations of this study.

Our decision to include only male subjects was primarily driven by the need to control for substantial metabolic variability arising from hormonal fluctuations associated with gender and menstrual cycles. Such a design is both common and necessary during the initial stages of establishing methodological frameworks and exploring mechanisms within exercise metabolomics (e.g., Maher, A. D., et al. (2010), Reue, K., & Wende, A. R. (2021)). This approach aim to first delineate the core metabolic response profiles induced by aerobic and resistance exercise within a relatively homogeneous metabolic cohort. This establishes a reliable baseline for subsequent studies in more complex populations, such as females and different age groups.

We have reinforced this point in the “Limitations” section of the manuscript. We explicitly state that the present findings apply only to healthy young males. Furthermore, we call for future studies specifically addressing women and gender-related metabolic differences, which is considered a crucial step towards advancing personalised exercise prescription.

c) This study did not employ a crossover design for the sequence of exercises. It must be acknowledged that potential sequence effects and residual effects exist. We compare metabolic profiles before aerobic and resistance exercises. The PCA plots show the two groups largely overlapping, indicating minimal differences in metabolic characteristics between samples. Following a recovery period of 48h or more after aerobic exercise, human metabolism has largely returned to pre-exercise levels.
d) We acknowledge that the lack of quantitative monitoring of diet and daily activities constitutes another limitation of this study. However, we have adopted a standardised protocol to minimise its impact.

We have expanded the relevant description in the Methods section: Within 24 hours before and after each exercise session, all participants were required to: abstain from alcohol and caffeine intake; avoid any moderate-to-vigorous physical activity; and consume standardised dinners and breakfasts. These measures aim to minimise individual variability in pre-test metabolic baseline.

5 Urine concentration varies considerably between individuals. We fully acknowledge that correcting for concentration variability is essential for accurate data interpretation. However, due to sample limitations, we are unable to obtain additional urine samples for measuring creatinine or specific gravity. We recognise this as a limitation of the present study. To minimise the impact of urine concentration variation on results, we implement the following measures:
  a) Standardised sample preparation: We adhere to a highly standardised sample preparation protocol, employing identical initial volumes and processing steps for all samples;
  b) Data-driven normalisation: During data preprocessing, we apply total signal normalisation to correct for overall signal intensity differences between samples;
  c) QC sample validation: QC samples are incorporated to monitor and ensure the stability of the entire analytical workflow. QC samples cluster tightly within the PCA plot.

Sample pretreatment procedures are listed in S1_File. S1_Fig1. shows the metabolomics total ion chromatogram (TIC). S1_Fig2. shows the PCA plots.

6 The results of the permutation test are shown in the figures. After 1,000 repetitions of permutation test, the models fit well.
7 Replace the p-values in the table with FDR adjusted p-values. Remove metabolites showing no significant change after correction.
8 Add HMDB IDs of the metabolites in the tables.
9 The descriptions for the figures and tables have been modified.
10 In this study, multiple amino acids (aspartic acid, glutamic acid, histidine, tryptophan, etc.) exhibit significant changes in levels before and after the two exercise modes, with metabolic pathway analysis results also focusing on amino acid metabolism. The discussion section mainly focuses on the differing trends in the same metabolite before and after two modes of exercise. Alternatively, the specific activation of a metabolite or pathway during one exercise section. The discussion is divided into four paragraphs:
a) The differing trends in changes in aspartic acid/asparagine before and after exercise in two modes;
b) Significant changes in glutamate/glutamine levels before and after resistance exercise;
c) The differing trends in histidine levels during the 24-hour recovery period following two modes of exercise;
d) Activation of the tryptophan metabolic pathway 24 hours after resistance exercise.

Speculative conclusions have been removed.

11 Acute exercise induces significant alterations in the metabolic characteristics of human urine. Two exercise modes promote exercise adaptation through specific amino acid metabolism. Aerobic exercise mobilizes aspartic acid, glutamic acid, and others to sustain a steady energy supply. In contrast, resistance exercise engages tryptophan and histidine, as well as their metabolites, to mediate immunosuppressive and antioxidant responses, thereby facilitating the transition from exercise-induced inflammation to a recovery state.
12 Future studies could expand sample sizes and increase sampling time points to achieve more accurate and comprehensive descriptions. Multi-omics approaches could be employed to cross-validate results and further refine biomarker selection. Comparative analyses could be conducted between individuals with different diseases or other populations and healthy males. Long-term exercise studies could also establish links between specific metabolic pathways and the effects of physical activity on organ function.
